# Perioperative selenium administration in cardiac surgery patients, a way out to reduce post surgical adversities? A meta analysis

**DOI:** 10.3389/fcvm.2023.1235247

**Published:** 2023-08-29

**Authors:** Syeda Tayyaba Rehan, Hassan ul Hussain, Laiba Imran, Farea Eqbal, Muhammad Sohaib Asghar

**Affiliations:** ^1^ Department of Medicine, Dow University of Health Sciences, Karachi, Pakistan; ^2^ Mayo Clinic-Rochester, Rochester, MN, United States

**Keywords:** AKI, cardiac surgery, meta analysis, oxidative damage, selenium

## Abstract

**Introduction:**

The oxidative damage suffered in cardiac surgery is associated with declining trace elements which lead to the development of multi organ dysfunction (MOD), acute kidney injury (AKI), or increased length of hospital stay (LOS). Recent evidence shows the cardioprotective role of the trace element selenium as it mitigates worsening outcomes post cardiac surgery. Hence, this meta analysis aims to investigate the role of selenium in lowering cardiac surgery related adverse outcomes.

**Methods:**

Literature search of five electronic databases was performed from the inception of the paper till 29th July, 2023. Eligibility criteria included; (a) randomized clinical trials with Adult patients (≥18 years) undergoing cardiac surgery (b) intervention with selenium pre or/and postoperatively; (c) a control group of a placebo, normal saline, or no selenium. Outcomes of interest include postoperative mortality, LOS in the hospital and Intensive Care Unit (ICU), AKI, troponin I, and Creatinine Kinase-MB (CK-MB). The Cochrane bias assessment tool was used to evaluate the risk of bias. Outcomes were pooled with the Mantel-Haenszel Random-effects model using Review Manager.

**Results:**

Seven RCTs with 2,521 patients and 65% of males were included in this paper. No noticable differences were observed between selenium and control groups in terms of postoperative AKI, mortality, LOS in hospital and ICU, troponin I, and CK-MB levels. All studies had a low risk of bias on quality assessment.

**Discussion:**

Our meta analysis demonstrated no discernible effects of selenium infusion on post operative complications among patients undergoing cardiac surgery. Further large scale multi centered studies comparing the protective role of selenium with combined therapy of other bioactive agents are needed to provide convincing explanations.

**Systematic Review Registration:**

PROSPERO Identifier: 424920.

## Introduction

Cardiac surgery and its outcomes are determined by various preoperative, intraoperative and postoperative factors that contribute to a higher morbidity rate as comparison to noncardiac surgeries (1). Increased oxidative stress (OS) is a significant contributor to the pathophysiology of cardiovascular disease and has been implicated in disease progression, the main culprit being reactive oxygen species (ROS) (2). This metabolic disturbance is worsened when patients undergo cardiac surgery and are subjected to the cardiopulmonary bypass (CP) pump which further increases the levels of lipid hydroperoxides and advanced oxidation protein products (3), predisposing patients to postoperative complications such as acute respiratory distress syndrome and acute kidney injury (AKI) (4).

Bioactive metabolites such as iron, copper, zinc, and selenium are important in regulating cell metabolism and their antioxidative properties may help prevent the progression of cardiovascular disease (5). The oxidative damage suffered in cardiac surgery is associated with declining trace elements which may predispose patients to develop multiple organ dysfunction (MOD) (6). Therefore, it can be postulated that supplementation with these minerals before cardiac surgery may help prevent postoperative complications leading to better outcomes for patients.

The trace element, selenium, has specifically shown a protective effect against oxidative stress (7) as it forms an essential component of our body's antioxidant defense mechanism. The inflammatory signaling pathways that modify ROS are modulated through selenium as it suppresses the nuclear factor-kappa B (NF-B) cascade, hence enhancing antioxidant capacity and reducing the generation of interleukins and tumor necrosis factor alpha (TNF-alpha) (8). A meta analysis conducted by Jenkins et al., demonstrates that adding selenium to the antioxidant mixture helps reduce the mortality rate in patients with cardiovascular disease. Moreover, removing selenium from the mixture led to a significantly increased risk of mortality, further highlighting the role of selenium in the antioxidant mix (9). Metabolic therapy with selenium was also noticed to decrease organ dysfunction (10) and myocardial damage in patients undergoing cardiac surgery (8). Recent evidence has established the role of selenium in reducing the hospital and ICU LOS in trauma patients, however, no concrete evidence exists that determines whether similar results are obtained in cardiac surgery patients (11).

Previously, pooled analyses have been conducted that explore the effects of other metabolic agents such as vitamin C in complex cardiac surgeries (12) but so far no conclusive results exist to demonstrate whether selenium depletion is the real culprit behind worsening postoperative outcomes in cardiac surgery patients. Hence, our meta-analysis aims to discover whether intervention with a bioactive agent, selenium, in patients undergoing cardiac surgery could help improve postoperative outcomes such as LOS, mortality, AKI, and troponin I and Creatinine Kinase-MB (CK-MB) levels.

## Methods

Our meta-analysis followed the Preferred Reporting Items for Systematic Review and Meta-Analysis (PRISMA) guidelines and was conducted within the framework laid out by the Cochrane collaboration (13). This systematic review and meta analysis has been registered on Prospero (ID: 424920). PRISMA checklist for this paper is provided in Supplementary Table 1.

### Data sources and search strategy

Pubmed, Google Scholar, ScienceDirect, ClinicalTrials.gov, and Cochrane CENTRAL were searched for literature from the inception of the paper till 29th July 2023. The following Medical Subject Headings (MESH terms) were incorporated into our search string: “selenium”, “selenium compounds”, “organoselenium”, “organoselenium”, “organoselenium compounds”, “cardiac surgery”, “cardiac operation”, “heart surgery”, “cardiothoracic surgery”, “coronary artery bypass grafting”, “cardiopulmonary bypass”. The complete search strategies used in each of the databases are provided in Supplementary Table 2. While performing the literature search, no location or time restrictions were applied and unpublished literature records were thoroughly searched to ensure that no material pertinent to our study had been omitted.

### Study selection and eligibility criteria

The retrieved articles from the literature search were transferred to the Mendeley Desktop 1.19.8 (Mendeley Ltd., Amsterdam, Netherlands) to scan for duplicates. Once the duplicates were removed, two independent reviewers (STR, FE) screened the articles and selected them if they matched with the PICO criteria of our study. In the initial study selection process, articles were chosen after reading their abstract and later were finalized after screening their full textThe following eligibility criteria was used to select studies: (a) randomized controlled trials (RCTs); (b) adult patients (≥18 years) undergoing cardiac surgery [CABG, valvular surgery, aortic surgery, left ventricular assist device (LVAD) implantation, intra aortic balloon pump]; (c) intervention with selenium pre or/and postoperatively; (d) control group of placebo, normal saline or no selenium; (e) outcomes of interest including postoperative mortality, LOS in the hospital and Intensive Care Unit, AKI, troponin I and CK-MB. Excluded articles included observational studies, commentaries, letters, and case reports. Articles in a language other than English and articles that administered other metabolites with selenium in intervention groups such as Vitamin C, Zinc, and Calcium were also excluded to exclusively see the effects of selenium on patients undergoing cardiac surgery. Studies evaluating the effects of selenium on surgeries other than cardiac procedures were also eliminated from our paper.

### Data extraction and quality assessment

Data on baseline characteristics such as study type, sample size, mean age, intervention, dosage, control, type of surgery, and primary outcomes were extracted into a Microsoft Excel sheet. Two reviewers (FE and STR) independently assessed the quality of the seven included RCTs. A third independent reviewer was consulted to resolve any discrepancies in the risk of bias assessments between the two review writers (LI). The risk of bias was assessed using the The Cochrane bias assessment tool (14) that categorized the risk of bias criteria as either low, high, or unclear on the basis of items described in the Cochrane Handbook for Systematic Reviews of Interventions (15). The RCTs were assessed on the basis of the following five categories: (i) selection bias (ii) performance bias (iii) detection bias (iv) attrition bias (v) reporting bias.

### Statistical analysis

The Mantel-Haenszel Random-effects model was used to pool the results using Review Manager (Version 5.3. The Cochrane Collaboration). Continuous data in our study were presented as mean difference and 95% confidence interval (CI) and to represent dichotomous data, odds ratios (OR) with 95% CI were employed. Heterogeneity was quantified using Higgins *I*^2^ statistics; a value of *I*^2^ = 25%–50% was considered statistically mild, 50%–75% as moderate, and >75% as severe heterogeneity. A *p*-value <0.05 was considered significant and this was consistent across all the studies.

## Results

**Figure 1:**
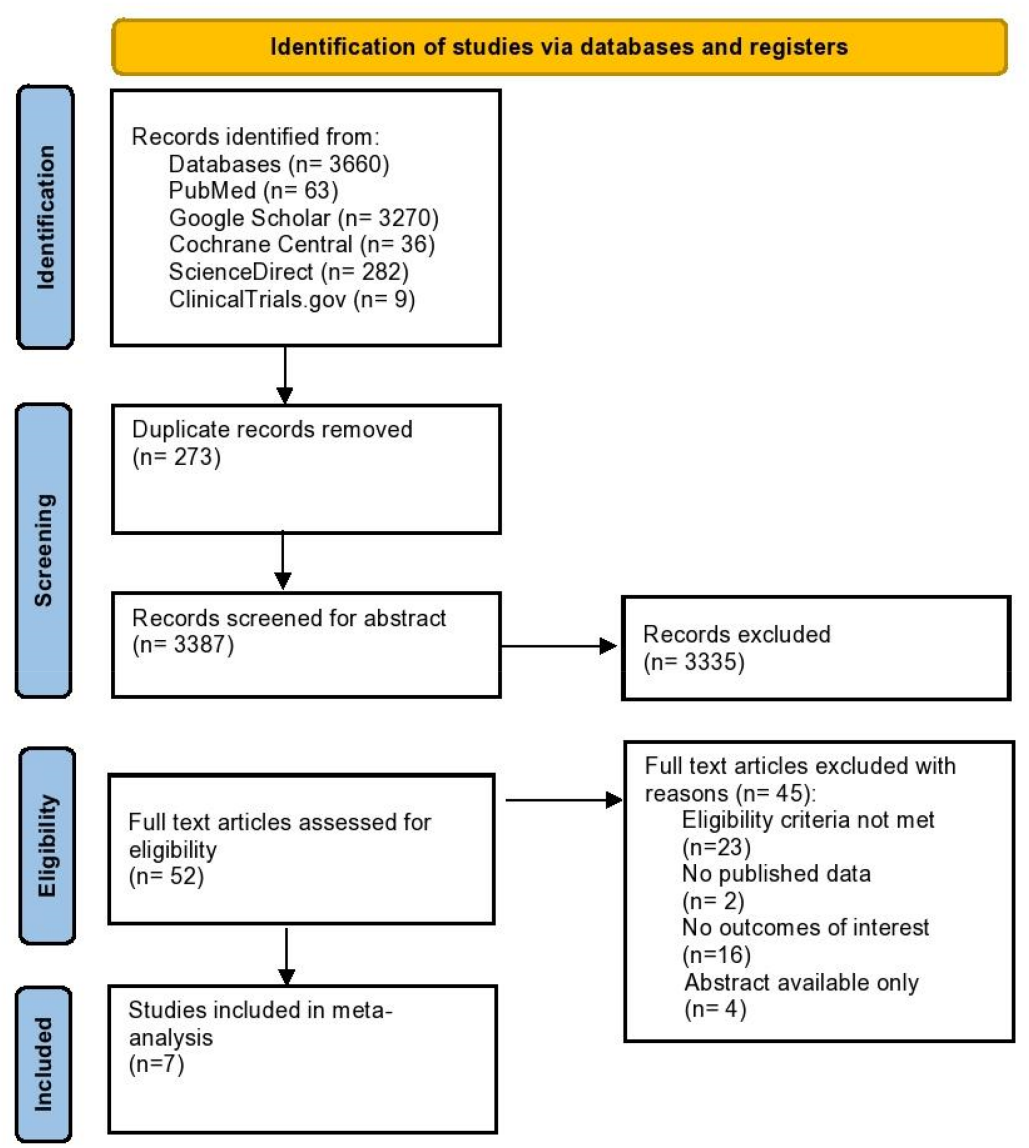
Prisma flow chart of literature search.

An initial search of 5 electronic databases and a few other sources provided 3,660 studies, out of which 3,387 records were left after removing the duplicate studies. 3,335 studies were eliminated after the title and abstract screening. After a full-text review of 52 articles which were assessed for their eligibility, 45 articles were excluded which did not meet the inclusion criteria. Consequently, 7 RCTs were finalized for this meta-analysis (16–22). A complete literature search has been highlighted in the PRISMA flowchart in Figure 1.

### Study characteristics, patients' baseline characteristics

Study characteristics and patients' baseline characteristics have been mentioned in detail in Tables 1, 2. A total of 2,521 patients were enrolled in these 7 RCTs, out of which 1,192 patients belonged to the selenium supplemented group, 1,194 patients belonged to the control group while 135 patients did not belong to any of our groups of interest. The mean age of the patients ranged from 52.8 years to 68.2 years with an average of 60.4 years. The percentage of males ranged from 38.3% to 95%, with a mean of 65.1% of the total population.

**Table 1:** Study characteristics.

Author (year)	Study design	Region	Dosage of selenium	Outcomes assessed	Type of surgery
Stoppe (16)	Randomized controlled trial	Canada, Germany	2,000 μg/L sodium selenite IV perioperatively, 2,000 μg/L postoperatively, 1,000 μg/L per day in ICU for 10 days	LOS (hosp + icu) AKI, Mortality	CABG (*n* = 907; 65.1%) Valvular intervention (*n* = 1,063; 89.7%) Surgical procedure at the isolated thoracic aorta (*n* = 2; 0.1%)
Laaf (19)	Randomized controlled trial	Germany	300 µg preoperative, IV bolus of 2,000 mcg selenium within 30 min of anesthesia, and 1,000 mcg selenium postoperative day 0 until day 13	Acute renal failure, Mortality, LOS	LVAD implantation (*n* = 20; 100%)
Ali (20)	Randomized controlled trial	Iran	500 µg of Se orally 14 and 2 h before surgery and every 12 h postoperatively for 2 days (overall 3,000 µg)	AKI, LOS (ICU stay)	Elective on-pump cardiac surgery (*n* = 120; 100%)
Masih (22)	Randomized controlled trial	Iran	1,000 µg sodium selenite	CK-MB and Troponin I levels immediately post op, 6 h, and 24 h after surgery	CABG (*n* = 67; 100%)
Amini (18)	Randomized controlled trial	Iran	0.5 mg twice daily from 24 h preoperatively until two postoperative days.	AKI, LOS (hospital + ICU, Mortality	Off pump CABG (137; 100%)
Sedighinejad (21)	Randomized controlled trial	Iran	IV bolus of 500 µg Se within 30 min through 48 h of hospitalization before the surgery and just before the induction of anesthesia.	CTnI and CK-MB measured before intervention and 6, 12, 24, and 48 h post op	CABG (*n* = 104, 100%)
Schmidt (17)	Randomized controlled trial	Switzerland	IV 4,000 µg selenium after induction of anesthesia and 1,000 µg/days selenium during ICU stay for 13 days.	LOS (hosp + icu), Mortality, AKI, Troponin	CABG (*n* = 218; 53.04%), Mechanical valve replacement (*n* = 63; 15.3%), Biological valve replacement (*n* = 157; 38.1%), Reconstructive valve surgery (*n* = 68; 16.5%), Aortic surgery (*n* = 64; 15.5%), Combined surgery (*n* = 130; 31.6%), With heart-lung machine (*n* = 363; 88.3%), Deep hypothermic cardiac arrest (*n* = 30; 7.2%), Intra-aortic balloon pump (*n* = 5; 1.2%)

ICU, Intensive care unit; LOS, Length of stay; AKI, Acute kidney injury; CABG, Coronary artery bypass grafting; LVAD, Left Ventricular assist device; CK-MB, Creatinine kinase-MB.

Se: Selenium; CTnI: Cardiac Troponin I.

**Table 2:** Population characteristics.

Author (year)	No. of participants (*n*)	Age [mean (SD)]	Sex	Comorbidities		Intervention (no. of participants)	Control (no. of participants)
				Heart disease	Diabetes		
Stoppe (16)	1,394	68.2 (10.4)	M = 1,043 (74.8%) F = 351 (25.2%)	Atrial fibrillation (21.3%) Previous MI (16.6%)	6%	Sodium selenite (*n* = 697)	Placebo (*n* = 697)
Laaf (19)	20	62.6 (9.4)	M = 19 (95%) F = 1 (5%)	Atrial fibrillation (30%) Previous MI (35%) Previous stroke (20%)	–	Selenium (*n* = 10)	Placebo (*n* = 10)
Ali (20)	120	52.8 (16.7)	M = 46 (38.3%), F = 74 (61.7%)	Previous MI (5%) Chronic heart disease (5%) Hypertension (38.3%)	20%	Selenium (*n* = 60)	Placebo (*n* = 60)
Masih (22)	200	56.4	M = 86 (43%), F = 114 (57%)	Hypertension (32.8%) Cerebral vascular attack (1.5%)	11%	Selenium (*n* = 100)	Normal saline (*n* = 100)
Amini (18)	272	59.3 (9.8)	M = 180 (66.2%) F = 92 (33.8%)	Previous MI (5.5%) Hypertension (39.7%)	36.3%	Selenium (*n* = 66)	No sodium selenite (*n* = 71)
Sedighinejad (21)	104	56.3 (8.9)	M = 68 (65.3%) F = 36 (34.7%)	Hypertension (57.6%)	49%	Selenium (*n* = 53)	Normal saline (*n* = 51)
Schmidt (17)	411	66.9 (10.5)	M = 302 (73.4%) F = 109 (26.5%)	Hypertension (62.7%) Coronary artery disease (57.6%) Atrial fibrillation (10.4%)	21.4%	Sodium selenite (*n* = 206)	Placebo (*n* = 205)

MI, myocardial infarction.

### Quality assessment

**Figure 2:**
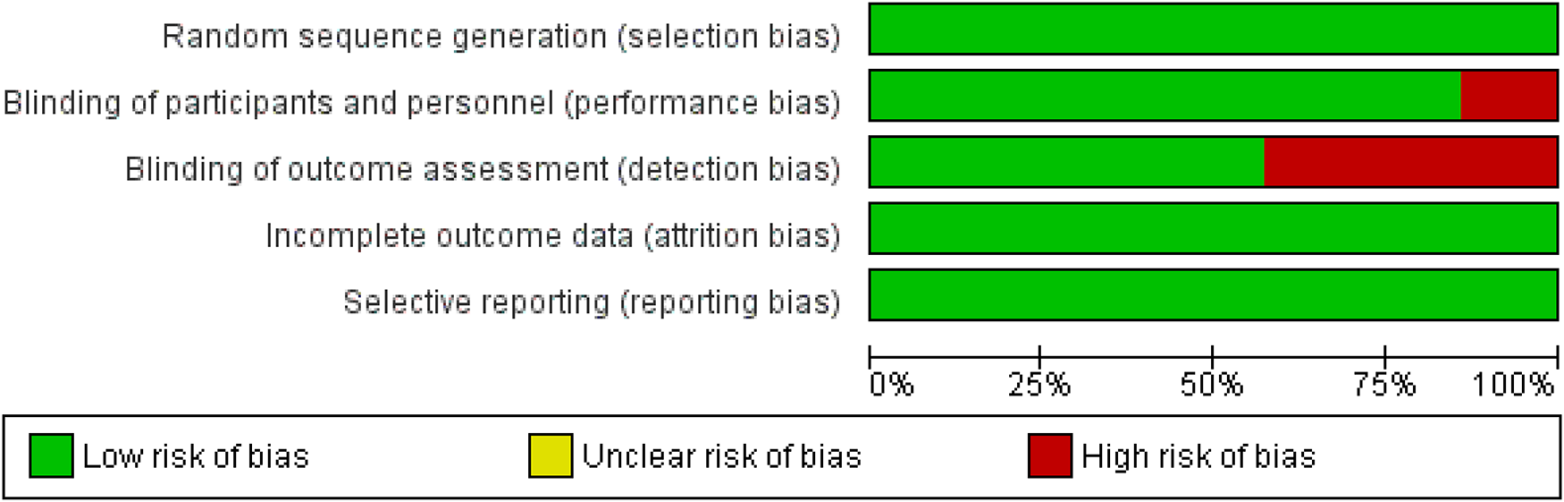
Risk of bias graph.

**Figure 3:**
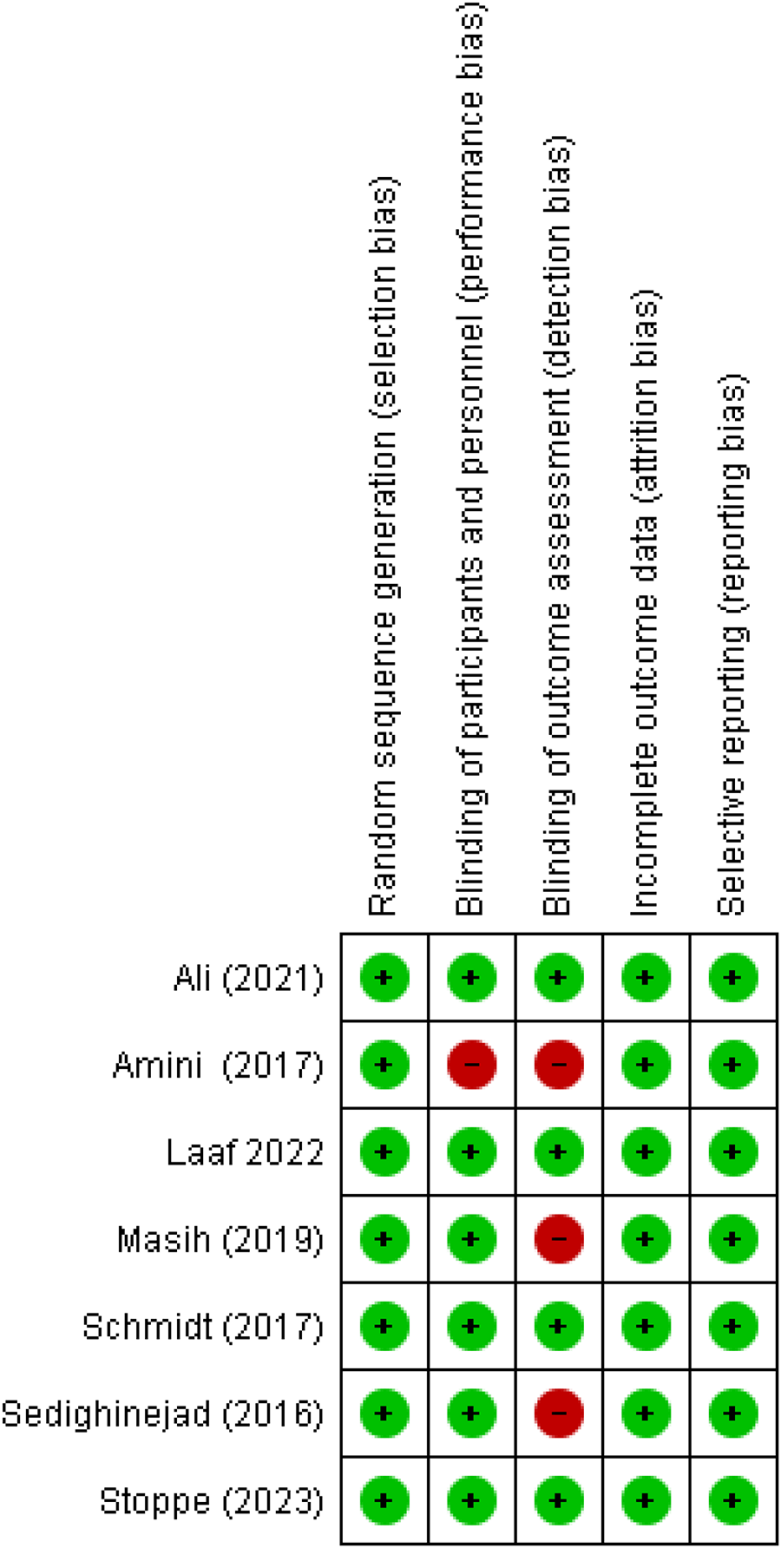
Risk of bias summary.

The majority of the included studies reported an overall low risk of bias, enhancing the authenticity of this meta analysis. Out of the 7 included studies, 3 studies reported detection bias which was the only main factor that hindered the quality of included studies (18, 21, 22). Additionally, the trial conducted by Amini et al. reported performance bias (18). On assessment, the overall quality of all the included studies was declared high. The detailed results of the quality assessment are listed in Figures 2, 3.

### Outcome analysis

**Figure 4:**
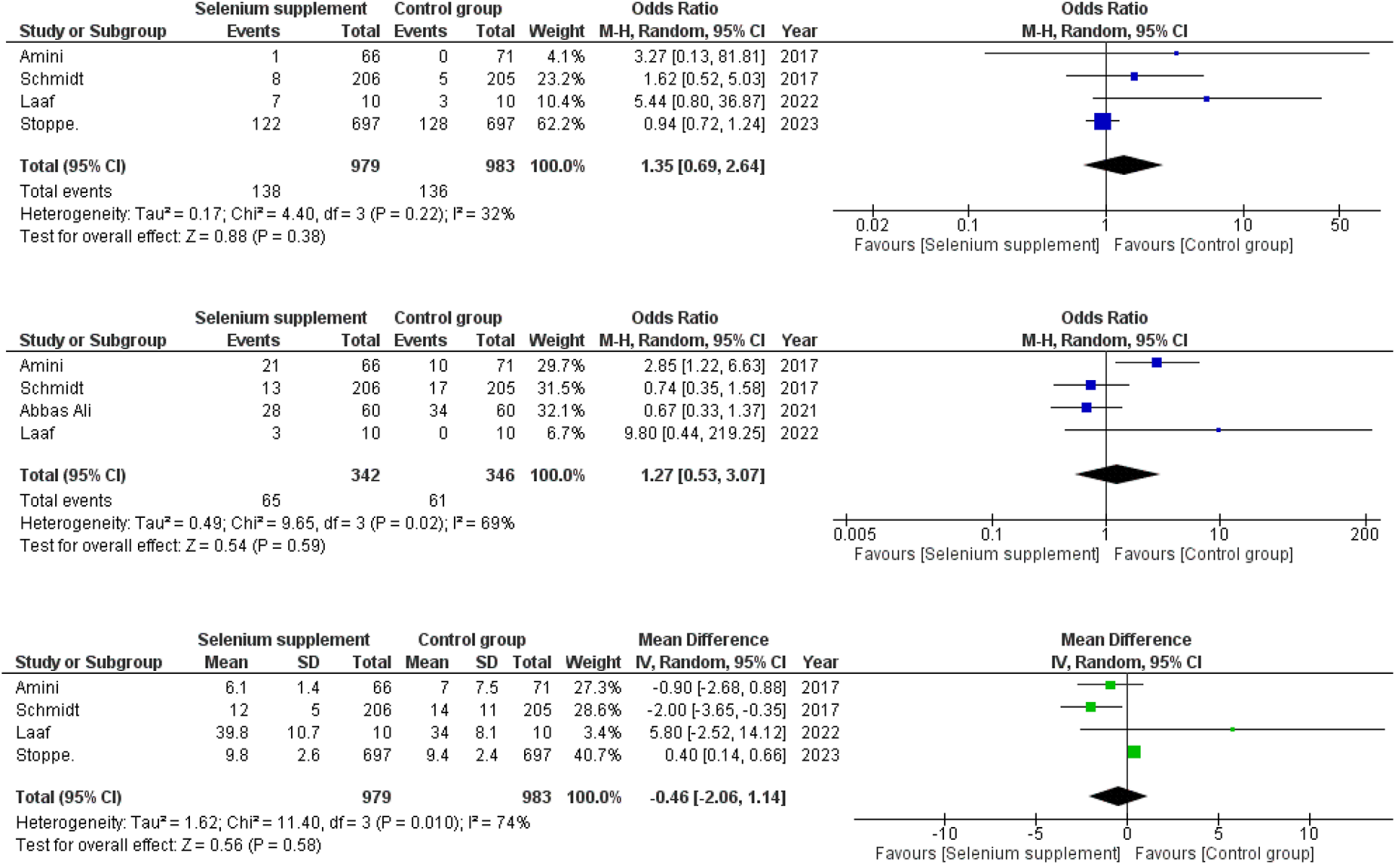
(**A**) Forest plot depicting odds ratios for mortality in selenium supplemented vs. control group patients. (**B**) Forest plot depicting odds ratios for AKI in selenium supplemented vs. control group patients. (**C**) Forest plot depicting mean differences for LOS in hospital in selenium supplemented vs. control group patients.

**Figure 5:**
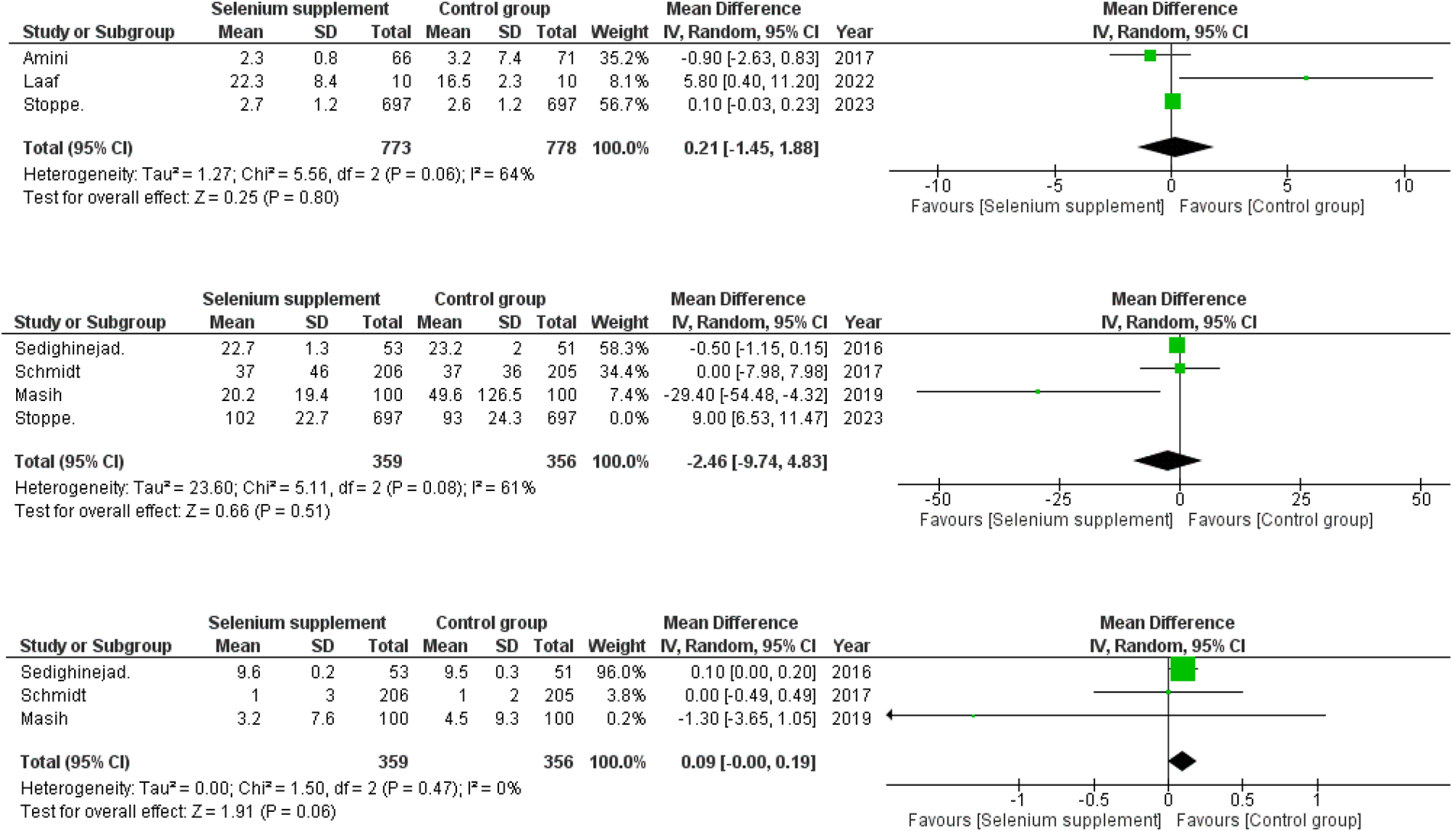
(**A**) Forest plot depicting mean differences for LOS in ICU in selenium supplemented vs. control group patients. (**B**) Forest plot depicting mean differences for post-operative CK-MB levels in selenium supplemented vs. control group patients after sensitivity analysis. (**C**) Forest plot depicting mean differences for post-operative Troponin I in selenium supplemented vs. control group patients.

The seven included RCTs (16–22) reported the role of selenium supplementation vs. control on postoperative outcomes in patients undergoing cardiac surgeries. Comprehensive forest plots with effect sizes of primary and secondary outcomes are given in Figures 4, 5.

### Mortality

Four RCTs (16–19) provided data for the effects of selenium supplementation on the mortality rate amongst the patients. A slight difference was observed between the two groups with higher chances of mortality in selenium supplementation group (OR =  1.35; 95% CI = 0.69–2.64; *P* = 0.38; *I*^2^= 32%) with a mild heterogeneity present amongst the RCTs (Figure 4).

### AKI

Four out of seven RCTs (17–20) provided data on the incidence of postoperative AKI. A minor difference was observed when the two groups were compared (OR =  1.27; 95% CI = 0.53–3.07; *P* = 0.59; *I*^2 ^= 69%) with mild chances of AKI in the selenium supplementation group. A moderate level of heterogeneity was observed between the RCTs (Figure 4).

### Hospital LOS

Four RCTs (16–19) reported data on post-operative hospital LOS in relation to selenium supplementation. A minor difference was observed between the two groups in terms of LOS in hospital (WMD = −0.46; 95% CI = −2.06–1.14; *P* = 0.58; *I*^2 ^= 74%) with the selenium supplemented group having a LOS some degree shorter than the control group. Only a moderate level of heterogeneity was found amongst the studies (Figure 4).

### ICU LOS

Three RCTs (16, 18, 19) published data on post-operative LOS in the ICU. A minor difference was observed amongst the two groups when their LOS in ICU were compared (WMD = 0.21; 95% CI = −1.45–1.88; *P* = 0.80; *I*^2 ^= 64%) with the selenium supplemented group having moderately longer ICU stay as compared to the non selenium group. Moderate level heterogeneity was found among the included studies (Figure 5).

### Postoperative CK-MB levels

Four of seven RCTs (16, 17, 21, 22) compared data on the post-operative CK-MB levels between selenium-supplemented and control group patients. A marginal difference was found in the postoperative CK-MB levels between the two groups (WMD = 0.73; 95% CI = −6.80–8.26; *P* = 0.85; *I*^2 ^= 95%). Considering the high level of heterogeneity, we performed sensitivity analysis by removing one study (16), (WMD = −2.46; 95% CI = −9.74–4.83; *P* = 0.51; *I*^2 ^= 61%) that greatly reduced the heterogeneity and showed lower postoperative CK-MB levels in selenium supplemented group (Figure 5).

### Postoperative troponin I levels

Out of seven RCTs, three (17, 21, 22) of them provided data on the post-operative troponin I levels between selenium-supplemented and control group patients. The pooled analysis showed a borderline difference between the two groups with mild elevation of troponin I levels in the selenium supplemented group postoperatively (WMD = 0.09; 95% CI = −0.00–0.19; *P* = 0.06; *I*^2 ^= 0%) (Figure 5). No in study heterogeneity was found during analysis (Figure 5).

## Discussion

In this comprehensive meta analysis we assessed the protective role of the bioactive agent, selenium, in the postoperative outcomes of patients undergoing cardiac surgery. Data from seven RCTs consisting of a total of 2,521 patients were pooled in this meta-analysis Our findings illustrate that administering selenium to patients undergoing cardiac surgery only slightly affected mortality, acute kidney injury, and LOS in hospital and ICU. Furthermore, individuals in the selenium group were demonstrated to have a greater risk of mortality, AKI and a longer ICU LOS as compared to the control group. Moreover, there was only a marginal statistical difference in the postoperative levels of cardiac biomarkers among the two comparable groups. Even though current literature has provided promising results regarding the benefits of selenium administration in cardiac surgery patients, our study found no firm data to validate the given hypothesis.

At present, there are several potential explanations that justify the benefits of selenium supplementation among patients undergoing cardiac surgery. A steep increase in OS, markedly reduced antioxidant (AOX) capacity, as well as postoperative inflammation has been observed among patients undergoing surgery (23). These factors not only lead to the development of organ failure but also increase mortality among such patients (24). According to evidence provided by current studies, selenium has been established as an essential element for improving heart functions and ameliorating heart injury (24). Selenium being a vital cofactor of various antioxidant enzymes could decrease the stress oxidative process and prevent postoperative complications after cardiac surgery (21, 25). Selenium in addition to other metals such as zinc and copper is vital for the functioning of the endogenous antioxidant system responsible for the degradation of free radicals produced as a result of various metabolic processes, thus maintaining a balance between oxidative stress and antioxidants available (9). Furthermore, it has been speculated that diminished selenium intake among patients with coronary heart disease and sepsis is a significant risk factor for an elevated post surgery mortality rate (26).

A wide variety of cohort and observational studies have evaluated the difference in the plasma levels of selenium pre and post cardiac surgery (7, 10, 17, 27). In a prospective observational trial of one hundred four cardiac surgical patients, a significant postoperative decline in circulating selenium levels was observed despite high-dose sodium-selenite administration prior to surgery (10). These cohort studies not only provided evidence regarding the drop in selenium levels post cardiac surgery but also demonstrated that low selenium levels are associated with severe complications post surgery (7, 10). It has been observed that the majority of the patients enrolled for cardiac surgery already have low selenium levels, implicating the pivotal role of this bioactive agent among these patients (6, 28). In a prospective observational clinical study, circulating selenium levels after completion of surgery were independently associated with the development of multi organ failure, peri operative inflammation and ICU length of stay (6). Additionally, Stevanovic et al. elucidated the importance of low postoperative selenium levels in predicting the likelihood of postoperative complications including organ dysfunction (29).

However, our meta analysis provided contrary findings regarding the protective role of selenium on post cardiac surgery complications. Several prospective studies in the past have illustrated that selenium supplementation was safe among cardiac surgery patients, but did not have any positive impact on post surgery clinical outcomes such as systemic inflammatory response and organ dysfunction (23, 30). This inconsistency in the findings between available data could be because of variable dosages of selenium supplementation among the different studies (10). Moreover, the limited sample size and the single-centered approach in the respective studies could also hinder the authenticity of the findings which should be interpreted with caution (23, 30). Further large scale multi centered studies are needed to provide a legitimate explanation for such ambiguity among findings of the protective role of selenium among patients undergoing cardiac surgery.

Vitamin C, another prominent antioxidant with distinctive organ-protective properties has also been evaluated for its cardioprotective effects. A meta analysis conducted to evaluate the correlation between peri operative vitamin C administration and overall outcomes after cardiac surgery revealed no significant effect on mortality (31). Furthermore, several meta analyses have assessed the protective role of iron supplementation on patients undergoing cardiac surgery (32–34). Yang et al. in their systematic review and meta analysis demonstrated no effect of iron supplementation on improved recovery of patients post cardiac surgery (31). Such corroboration with the results of previous meta analyses not only enhances the authenticity of our analysis but also highlights the ambiguity regarding the role of bioactive agents in postoperative outcomes of patients undergoing cardiac surgery, Furthermore, several studies (27, 35) have demonstrated the protective role of combined bioactive infusion rather than giving supplements separately. For example, a single centered trial aimed to investigate the impact of combined micronutrients supplementation (consisting of selenium, zinc, vitamin C, and vitamin B1), on clinical outcomes in ICU patients with conditions characterized by oxidative stress, revealed significant reductions in inflammatory response among the intervention group (35). Another similar study investigated the potency of peri operative metabolic therapy (consisting of coenzyme Q10, magnesium orotate, lipoic acid, omega-3 fatty acids, and selenium) among cardiac surgery patients (27). This study illustrated that such metabolic therapy is not only associated with enhanced redox status but is also linked with diminished myocardial damage and shortened length of hospital stay (27). Such consistency among the available studies could be employed to elucidate the protective role of a combined bioactive therapy rather than administering the bioactive agents separately.

It is imperative to note that the numerous benefits associated with selenium intake depend on its concentration and chemical structure. It has been highlighted by several studies that inorganic selenium and the daily ingestion of selenium exceeding 1 mg per 1 kg of body can be toxic to an average person (36). The recommended intake of selenium by the World Health Organization (WHO) was 30–40 mg per day for adult men and women (36). The main organic forms of selenium are selenomethionine (Semet) and selenocysteine (Secys), an essential component of selenoprotein which play a key role in numerous body functions including antioxidation (37). There has been increasing evidence regarding the risk of developing hyperglycemia and hence type 2 diabetes mellitus (T2DM) in addition to developing dyslipidemia among individuals with high selenium levels (11). Nevertheless, some studies have also illustrated the neutral or positive effect of selenium on T2DM. Therefore, further research is needed to clarify the potential benefits and adverse outcomes associated with different compositions of selenium in order to maximize its benefits among critical patients.

### Strengths and limitations

Until yet, no meta analysis has evaluated the protective role of the bioactive agent, selenium, on the post operative outcomes of patients undergoing cardiac surgery. Our meta analysis is the first pooled result of comparative trials of selenium vs. no selenium or placebo group in improving postoperative outcomes of cardiac surgery. The in-depth search approach employed in this study yielded only those studies that exclusively evaluated the protective role of selenium alone rather than infusing it in combination with other bioactive agents. The randomized, double or single-blinded, and placebo-controlled approach as well as the low risk of bias of the studies included in this comprehensive meta analysis further strengthens the credibility of our pooled analysis. However, a few limitations have hindered the authenticity and the acceptability of the results of this study. First, the comparatively moderate sample size and heterogeneity of the included population may not only limit the statistical power of the paper but may also prevent the results from being extrapolated in the general population. Another major limitation of this study was the variation in the dosages of selenium supplementation in the different RCTs included in our review and hence an absence of dose response analysis which could hinder the authenticity of the findings.

## Conclusion

In conclusion, our meta analysis demonstrated no discernible effect of selenium infusion on post operative complications among patients undergoing cardiac surgery. Considering the crucial role of bioactive agents in post operative recovery of cardiac surgery patients, it has become vital to shift the focus of research towards bioactive metabolites such as selenium in reducing post operative mortality and morbidity. Further large scale multi centered studies comparing the protective role of selenium with combined therapy of bioactive agents are needed to provide an authentic explanation for the effectiveness of selenium that could be extrapolated in the general population.

## Data Availability

The original contributions presented in the study are included in the article/**Supplementary Material**, further inquiries can be directed to the corresponding author/s.
